# Inflammation impairs fertility and oocyte quality in systemic lupus erythematosus by triggering oxidative stress-mediated ferroptosis

**DOI:** 10.1038/s41419-026-09028-x

**Published:** 2026-06-30

**Authors:** Ruolin Mao, Juepu Zhou, Xiangfei Wang, Rui Long, Meng Wang, Hong Chen, Nan Xiao, Qingsong Xi, Lei Jin, Lixia Zhu

**Affiliations:** 1 Reproductive Medicine Center, Tongji Hospital, Tongji Medical College, Huazhong University of Science and Technology, Wuhan, China; 2 National Clinical Research Center for Obstetrics and Gynecology, Tongji Hospital, Tongji Medical College, Huazhong University of Science and Technology, Wuhan, China; 3 Department of Center for Reproductive Medicine, Tianjin Central Hospital of Obstetrics and Gynecology, Tianjin, China; 4 Nankai University Affiliated Maternity Hospital, Tianjin Key Laboratory of Human Development and Reproductive Regulation, Tianjin, China

**Keywords:** Disease model, Mechanisms of disease, Chronic inflammation

## Abstract

Systemic lupus erythematosus (SLE) is an autoimmune disorder associated with impaired female fertility, as suggested by our previous clinical cohort data. However, the intrinsic mechanisms underlying oocyte quality deterioration remain incompletely understood. Using a chronic SLE mouse model, we show that SLE-associated inflammation is accompanied by impaired oocyte maturation, reduced developmental competence, and mitochondrial dysfunction, together with increased oxidative stress and ferroptosis-related alterations, an iron-dependent process characterized by lipid peroxidation. Pharmacological interventions revealed that both hydroxychloroquine and melatonin attenuated inflammation- and oxidative stress–associated abnormalities and partially improved oocyte developmental outcomes. In addition, inhibition of ferroptosis using ferrostatin-1 ameliorated ferroptosis-associated alterations and partially restored meiotic maturation and oocyte quality. Together, these findings establish a mechanistic link between SLE-induced inflammation, oxidative stress, ferroptosis, and impaired oocyte developmental competence. These results provide a framework for understanding inflammation-associated oxidative stress and ferroptosis-related alterations in SLE-associated reproductive dysfunction and may inform future therapeutic exploration.

**Figure:**
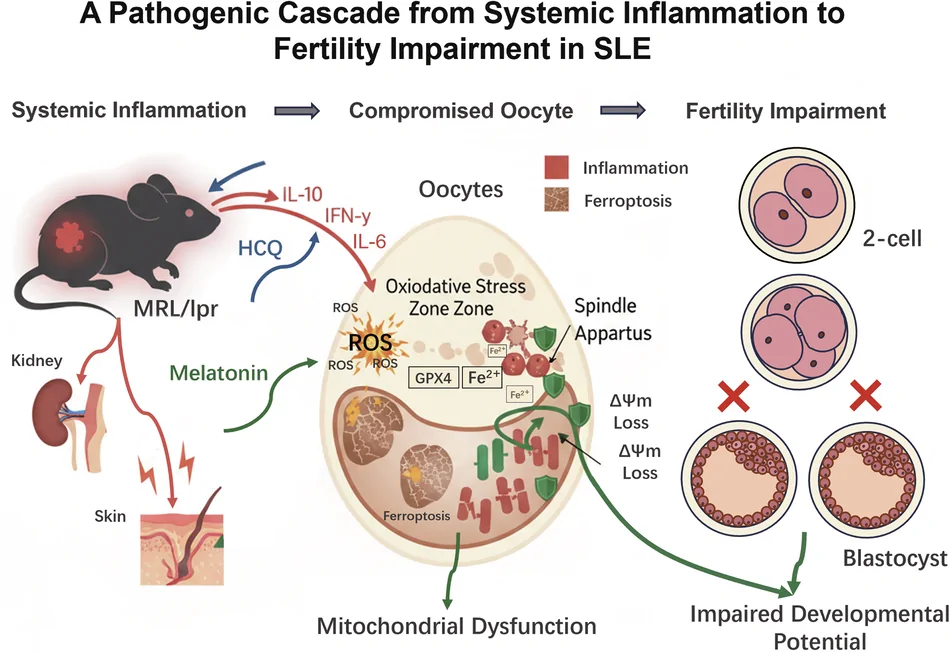

## Introduction

Systemic lupus erythematosus (SLE) is a chronic autoimmune disorder characterized by loss of immune tolerance, leading to the production of autoantibodies against nuclear antigens and immune complex–mediated multi-organ inflammation [1]. Despite extensive investigation, its etiology remains incompletely understood due to clinical heterogeneity and complex serological features [2]. Notably, SLE predominantly affects women of reproductive age, with a global prevalence exceeding 5 million individuals [3], raising increasing concerns regarding reproductive health in this population.

Previous studies have largely focused on pregnancy-related complications in women with SLE [4, 5], including increased risks of miscarriage, hypertensive disorders, and lupus nephritis, particularly in patients with active disease. However, the impact of SLE on female fertility itself remains controversial. While some studies report comparable ovarian reserve between SLE patients and healthy controls [6, 7], others suggest impaired fertility [8, 9], and reduced family size [10]. In addition to biological factors, reduced fertility intentions and delayed childbearing may further contribute to these observations [11]. These discrepancies highlight the need to clarify the biological effects of SLE on female fertility.

From a genetic perspective, our previous study demonstrated a causal relationship between SLE and primary ovarian failure (POF) using a two-sample Mendelian randomization approach [12]. Consistently, our retrospective cohort analysis revealed an association between SLE and impaired female fertility, including reduced oocyte competence and compromised embryo development [13]. These findings suggest that oocyte quality may be a critical determinant of reproductive impairment in SLE. However, the underlying mechanisms remain poorly understood.

Inflammation is a central pathological feature of SLE and contributes to multi-organ damage. SLE-associated inflammation has been implicated in autoimmune oophoritis and other reproductive abnormalities [14, 15]. Moreover, several immune-mediated inflammatory diseases, including rheumatoid arthritis [16], inflammatory bowel disease [17], Addison’s disease [18], and primary Sjögren syndrome [19], have been associated with impaired female fertility, particularly during active disease. These observations support a role for chronic inflammation in reproductive dysfunction, although the downstream cellular mechanisms linking inflammation to oocyte damage remain unclear.

Emerging evidence indicates that oxidative stress is a key downstream consequence of inflammation and a major contributor to oocyte dysfunction. Excessive reactive oxygen species (ROS) can disrupt mitochondrial function, impair meiotic progression, and reduce developmental competence. In addition, ferroptosis [20], an iron-dependent form of regulated cell death driven by lipid peroxidation, has recently been implicated in reproductive disorders and may represent a potential downstream mechanism linking oxidative stress to oocyte damage.

In the present study, we established a chronic SLE mouse model to investigate how SLE-associated inflammation affects oocyte quality and female fertility. We focused on the relationship between inflammatory status, oxidative stress, mitochondrial dysfunction, and ferroptosis-related alterations, and further evaluated potential therapeutic interventions, including hydroxychloroquine, melatonin, and ferroptosis inhibition, to determine whether these pathways can be targeted to improve oocyte quality and reproductive outcomes in SLE.

## Materials and methods

### Ethics statement

Animal procedures were approved by the Institutional Animal Care and Use Committee (IACUC) of Tongji Medical College, Huazhong University of Science and Technology (IACUC Number 4106). All experiments were conducted in accordance with the Guide for the Care and Use of Laboratory Animals.

### Animals

MRL/lpr mice were purchased from Shulaibao (Wuhan) Biotechnology Co., Ltd. (Wuhan, China) as SLE model group [21], and female Institute of Cancer Research (ICR) mice were purchased from Cyagen Biosciences (Suzhou, China) as controls. Female mice aged 8–16 weeks were used for oocyte collection, fertility assessment, ovarian histology, and intervention experiments unless otherwise specified. For longitudinal analyses, serum samples were collected from 4 to 20 weeks of age, and oocyte ROS levels were assessed from 4 to 16 weeks of age. For tissue, oocyte, and sperm collection, mice were euthanized by CO₂ inhalation followed by cervical dislocation, in accordance with institutional guidelines for animal care and use.

### Mouse oocytes collection and culture

Mice were euthanized prior to ovary dissection for GV oocyte collection. GV oocytes were collected by puncturing antral follicles with a fine needle under a dissecting microscope. The oocytes were cultured in M2 medium (Aibei Biotechnology, Nanjing, China) for 16 h at 37 °C under 6% CO₂, 5% O₂, and 89% N₂ for in vitro maturation. The germinal vesicle breakdown (GVBD) rate was calculated 2 hours after in vitro culture, while the maturation rate was assessed at 12, 14, and 16 hours, respectively. For ovulated oocytes collection, female mice were injected with 10 IU of PMSG, followed by 10 IU of hCG 48 hours later. Oocytes were collected from the oviductal ampullae approximately 13–14 hours after hCG injection.

### In vitro fertilization and embryo culture

Matured oocytes were fertilized in vitro with sperm isolated from the cauda epididymides. Male mice were euthanized prior to sperm collection. Successful fertilization was confirmed by the presence of two pronuclei (2PN). Zygotes were cultured in KSOM medium (Aibei Biotech, China) in microdroplets overlaid with mineral oil and incubated at 37 °C under 6% CO₂, 5% O₂, and 89% N₂ for 5–6 days.

### Offspring collection

To obtain offspring through natural delivery, female mice were paired with stud males in a 1:2 ratio in the same cage. Successful mating was confirmed by the presence of vaginal plugs. Offspring were identified by sex and housed for 19–21 days post-delivery.

### Melatonin treatment

For in vitro experiments, GV oocytes from CON and SLE-M mice were cultured in M2 medium containing 1 μmol/L melatonin (HY-B0075, MedChemExpress, USA) [22–24]. For in vivo experiments, female mice were administered daily oral doses of melatonin (30 mg/kg body weight) for 27 days, based on previous studies [22, 25] and our preliminary experiments.

### Hydroxychloroquine (HCQ) treatment

For in vivo experiments, female mice were administered daily oral doses of HCQ (50 mg/kg body weight) for 27 days (HY-B1370, MedChemExpress, USA), following the dosage outlined in the literature [26, 27] and based on our preliminary data.

### Ferrostatin-1 treatment

For ferroptosis inhibition experiments, GV oocytes from CON and SLE-M mice were cultured in M2 medium supplemented with ferrostatin-1 (Fer-1, HY-100579, MedChemExpress, USA) at a final concentration of 5 μmol/L [28]. Oocytes were cultured under standard in vitro maturation conditions and subsequently used for analysis of meiotic maturation, redox status, mitochondrial function, and ferroptosis-associated indicators.

### 24-hour urine protein quantification

Urine was collected using mouse metabolic cages and stored at −80 °C for later analysis. Urinary protein concentration was determined using the Coomassie Brilliant Blue (CBB) method (C035-2-1, Nanjing Jiancheng Bioengineering Institute).

### H&E staining and immunohistochemistry

For histological examination, mouse kidneys and ovaries were fixed in 4% paraformaldehyde and dehydrated through a graded ethanol series at room temperature. After dehydration, tissues were embedded in paraffin, and serial sections (2 μm thickness) were prepared.

For hematoxylin and eosin (H&E) staining, sections were stained with hematoxylin (31890610, Sigma, USA) and eosin (HHS16, Sigma, USA) according to standard protocols.

For immunohistochemistry (IHC), paraffin sections were deparaffinized, rehydrated, and subjected to antigen retrieval in citrate buffer (pH 6.0). Endogenous peroxidase activity was blocked with 3% hydrogen peroxide, followed by blocking with normal serum. Sections were then incubated overnight at 4 °C with primary antibodies against CD3 and CD68. After washing, sections were incubated with appropriate HRP-conjugated secondary antibodies, and immunoreactivity was visualized using diaminobenzidine (DAB). Nuclei were counterstained with hematoxylin. Whole-slide images were acquired using a Pannoramic 250 FLASH III digital slide scanner (3DHISTECH, Hungary) under identical scanning settings.

Quantification of staining intensity was performed using ImageJ software by calculating the mean optical density (MOD) of DAB staining in selected regions. At least three non-overlapping fields per section were analyzed. All analyses were conducted in a blinded manner

### ELISA

Blood samples were collected from mice at 2-week intervals from 4 to 18 weeks of age via retro-orbital sinus puncture under anesthesia. Samples were allowed to clot at room temperature for 30 min and then centrifuged at 3000 rpm for 10 min at 4 °C to separate the serum. The serum was collected and stored at −80 °C until further analysis.

Serum concentrations of IL-6 (JM-02446M2-JK), IL-10 (JM-02459M1-JK), IFN-γ (JM-02465M1-JK), and anti-dsDNA (JM-02493M1) were measured using ELISA kits (JINGMEI Biotechnology, China) according to the manufacturer’s instructions. Additionally, the levels of anti-Müllerian hormone (AMH), follicle-stimulating hormone (FSH), luteinizing hormone (LH), and estradiol (E2) were assessed following the protocols provided with the respective ELISA kits (JINGMEI Biotechnology, China).

### Quantification of ovarian follicles

To quantify ovarian follicles, ovaries were serially sectioned at 4 μm thickness across the maximum ovarian plane, and every fifth section was stained with hematoxylin for morphological evaluation. Follicles at different developmental stages, including primordial, primary, secondary, antral, and atretic follicles, were identified and counted according to established morphological criteria [29]. Follicle numbers shown in the figures represent the cumulative counts from the analyzed serial sections rather than a single section. Follicle counting was performed independently by two investigators blinded to group allocation.

### Micro-transcriptome sequencing and RNA library construction

RNA libraries were constructed using the Smart-Seq2 method, as previously described [30]. Sequencing was performed on an Illumina NovaSeq 6000 platform by Annoroad Gene Technology (Beijing, China). RNA was extracted and purified from 20 to 30 GV oocytes per sample. Differentially expressed genes (DEGs) were identified using DESeq2, with thresholds of |fold change | > 1.5 and *P* < 0.05. DEG expression was visualized using a volcano plot and heatmap generated by the “EnhancedVolcano” and “pheatmap” packages, respectively. Gene ontology (GO) analysis, pathway enrichment analysis, and Gene Set Enrichment Analysis (GSEA) were conducted using the “clusterProfiler” package.

### Measurement of cytoplasmic NADPH and GSH levels

NADPH, NADP + , GSH, and GSSG levels in mouse oocytes were measured using assay kits (Beyotime Biotechnology, China) according to the manufacturer’s instructions.

### Measurement of serum T-SOD, MDA, and GSH-PX

Serum levels of T-SOD, MDA, and GSH-PX were quantified using assay kits (Beyotime Biotechnology, China), following the manufacturer’s guidelines.

### RNA Isolation and quantitative RT-PCR (qRT-PCR)

Total RNA was extracted from pooled oocytes (*n* = 30) using TRI reagent (Sigma-Aldrich, T9424) as per the manufacturer’s protocol. Reverse transcription was performed using the Hifair III 1st Strand cDNA Synthesis Kit (Yeasen, 11139ES60) following the manufacturer’s instructions. Quantitative RT-PCR was carried out using SYBR Green Master Mix (Yeasen, 11203ES08) on the Quantagene q225 qPCR system, according to the manufacturer’s instructions. Relative mRNA levels were normalized against values from Gapdh mRNA, and quantification of fold change was determined by the comparative Ct method. Primer sequences are listed in Supplementary Table (Supplementary Information).

### TUNEL assay

The terminal deoxynucleotidyl transferase dUTP nick end labeling (TUNEL) assay was performed on blastocysts using the TMR (red) Cell Apoptosis Detection Kit (G1502, Servicebio, China) following the manufacturer’s protocol. Blastocysts mounted on glass slides were imaged using a LSM 900 confocal microscope (Carl Zeiss, Germany). TUNEL-positive cells and total cell numbers were counted from acquired fluorescence images using ImageJ software. The percentage of apoptotic cells was calculated as the number of TUNEL-positive cells divided by the total number of cells in each blastocyst. Cell counting was performed independently by two investigators blinded to group allocation.

### Immunofluorescence and morphological assessment

Immunofluorescence staining of oocytes and blastocysts was performed as previously described [31]. Briefly, samples were fixed, permeabilized, and blocked before incubation with the following primary antibodies: anti-GPX4 (67763-1-Ig, Proteintech, China), anti-β-tubulin (#2146, Cell Signaling Technology, USA), anti-OCT3/4 (sc-5279, Santa Cruz Biotechnology, USA), anti-CD3 (81324-1-RR, Proteintech, China), and anti-CD68 (66231-2-Ig, Proteintech, China). After washing, appropriate secondary antibodies were applied. Chromosomes were counterstained with Hoechst 33342(CM12592, Proteintech, China) for 10 minutes. The following secondary antibodies were used: FITC-labeled goat anti-rabbit (A0562, Beyotime, China) and Cy3-labeled goat anti-mouse (A0521, Beyotime, China). Images were captured using a Zeiss LSM900 confocal microscope (Carl Zeiss, Germany) and processed with ZEN 3.9 software. Morphological and image-based evaluation of oocytes, including cytoplasmic appearance, polar body morphology, zona pellucida regularity, chromosome alignment, and spindle morphology, was performed independently by two investigators blinded to group allocation. Oocytes with homogeneous and evenly distributed cytoplasm were classified as normal, whereas those exhibiting coarse or uneven cytoplasmic granularity were classified as having granular cytoplasm. Abnormal polar body morphology was defined as a fragmented, enlarged, irregularly shaped, or degenerated first polar body. Irregular zona pellucida was defined as an uneven contour or visibly non-uniform thickness. Chromosome misalignment was defined as one or more chromosomes clearly displaced from the metaphase plate. Aberrant spindle morphology was defined as loss of the typical bipolar barrel-shaped structure, including disorganized, elongated, multipolar, or asymmetric spindle configurations. For blastocyst analysis, total cell number and OCT4-positive inner cell mass (ICM) cell number were counted from acquired fluorescence images using ImageJ software. Quantification was performed independently by two investigators blinded to group allocation.

### Assessment of ROS, mitochondrial function, and ferroptosis in oocytes

Intracellular reactive oxygen species (ROS) levels were detected using carboxy-H₂DCFDA (S0033S, Beyotime, China) according to the manufacturer’s instructions. Mitochondrial distribution was visualized with MitoTracker Red (C1049B, Beyotime, China), and mitochondrial membrane potential (ΔΨm) was assessed using the JC-1 Assay Kit (C2006, Beyotime, China). To evaluate ferroptosis-related parameters, FerroOrange (HY-D1533, MedChemExpress, USA), BODIPY™ 581/591 C11 (S0043S, Beyotime, China), and monochlorobimane (HY-101899, MedChemExpress, USA) were used according to the manufacturers’ instructions. All fluorescence images were acquired using identical laser power, gain, and exposure settings within each experiment on a Zeiss LSM900 confocal microscope (Carl Zeiss, Germany) and analyzed using ImageJ software (NIH, USA) [32]. For ROS, lipid peroxidation, Fe²⁺, GSH, and GPX4 analyses, the fluorescence intensity of each individual oocyte was measured within a manually defined region of interest encompassing the ooplasm, and the mean fluorescence intensity after background subtraction was used for quantification. For mitochondrial analysis, mitochondrial distribution patterns were evaluated based on MitoTracker Red fluorescence. Oocytes were classified as exhibiting either normal mitochondrial distribution or evident mitochondrial aggregation according to fluorescence localization patterns. The aggregation ratio was calculated as the percentage of oocytes showing mitochondrial aggregation within each independent experiment. For JC-1 staining, mitochondrial membrane potential was evaluated by calculating the red/green fluorescence intensity ratio. All image analyses were performed in a blinded manner.

### Statistical analyses

Statistical analyses were performed using SPSS 26 (IBM, USA) and GraphPad Prism 9. Data are presented as mean ± SD. Comparisons between two groups were performed using unpaired two-tailed Student’s *t*-test. Comparisons among multiple groups were conducted using one-way ANOVA followed by Tukey’s multiple comparison test. A *P* < 0.05 was considered statistically significant. For oocyte-based assays, *n* represents the number of oocytes analyzed, derived from at least three independent biological replicates. For embryo assays, *n* represents the number of embryos analyzed from independent IVF experiments. For in vivo experiments, *n* represents the number of mice. The definition of *n* for each experiment is specified in the corresponding figure legends.

## Results

### SLE-M mice exhibit systemic autoimmunity and chronic inflammation

**Fig. 1:**
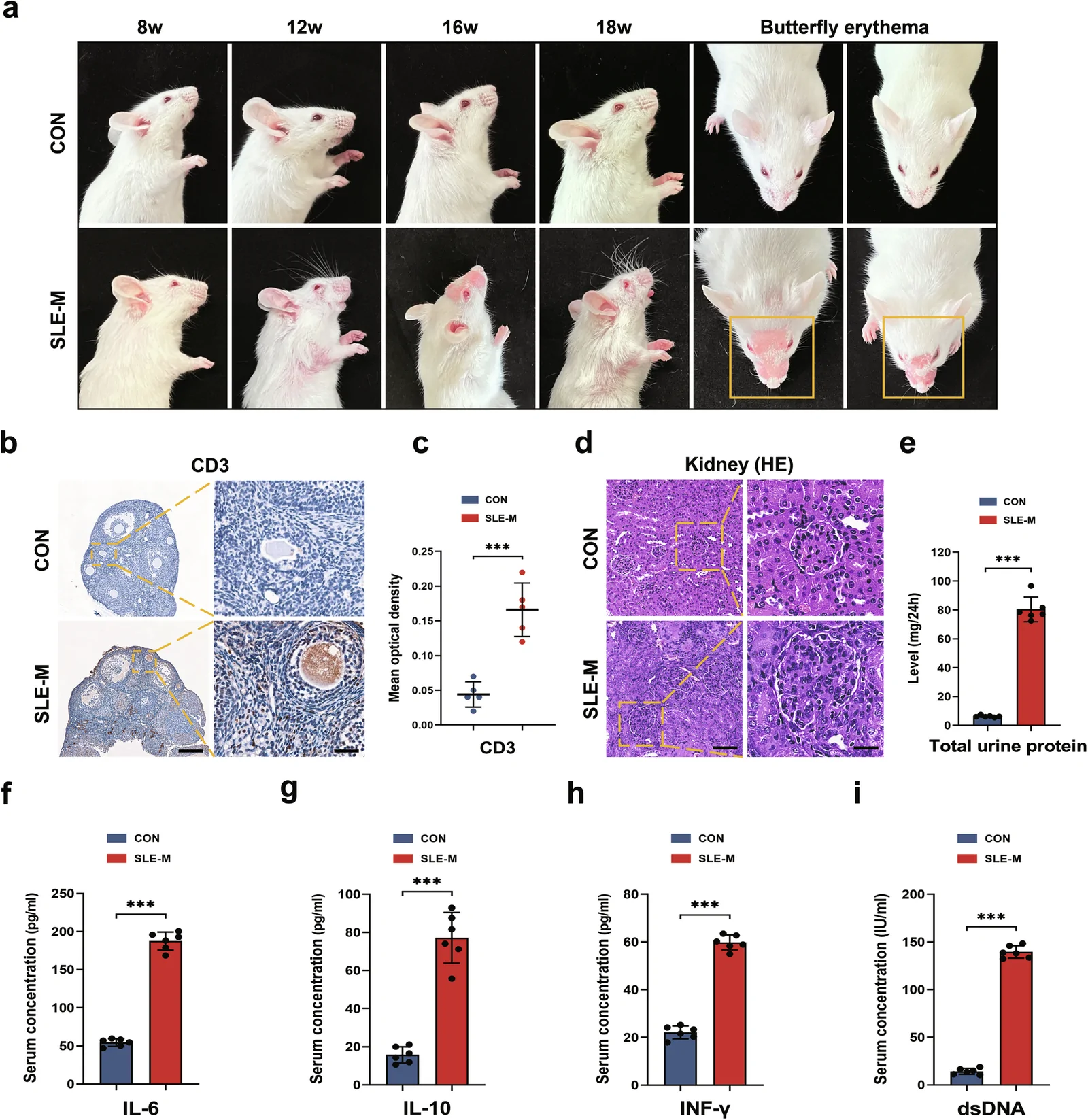
Lupus-like changes and characteristic elevated levels of inflammation were presented in SLE-M mice. **a** Representative images showing progressive skin lesions and characteristic butterfly erythema in SLE-M mice. **b** Representative immunohistochemical staining of CD3 in ovarian sections from CON and SLE-M group. Scale bar, 30 μm. **c** Quantification of CD3 staining intensity in ovarian tissues (*n* = 5 mice per group). **d** Representative H&E staining of kidney sections from CON and SLE-M mice. Scale bar, 100 μm. **e** 24-hour urinary protein levels (*n* = 6 mice per group). **f**–**i** Serum levels of IL-6, IL-10, IFN-γ and anti-dsDNA antibodies in CON and SLE-M group (*n* = 6 mice per group). Data are presented as mean ± SD. Statistical significance was determined using unpaired two-tailed Student’s *t*-test. *P < 0.05, **P < 0.01, ***P < 0.001.

The MRL/lpr mouse model (SLE-M) recapitulated key clinical features of systemic lupus erythematosus [21]. From 12 weeks of age, SLE-M mice developed characteristic facial lesions, presenting as erythematous patches accompanied by progressive alopecia (Fig. 1a). In addition to systemic manifestations, ovarian tissues exhibited increased immune cell infiltration. Immunohistochemical staining revealed increased CD3-positive T cells in SLE-M ovaries compared with controls (Fig. 1b, c). Consistently, CD68-positive cell infiltration was also elevated (Supplementary Fig. S2a-b), indicating an altered local ovarian immune environment. Histological examination of kidney tissues at 16 weeks showed marked pathological changes, including glomerular hypertrophy and inflammatory cell infiltration within both glomeruli and interstitium (Fig. 1d). These abnormalities were accompanied by significantly increased 24-hour urinary protein levels (Fig. 1e), indicating impaired renal function. Serum analysis further demonstrated significantly elevated levels of IL-6, IL-10, IFN-γ, and anti-dsDNA antibodies (Fig. 1f-i), supporting the presence of a systemic autoimmune and inflammatory state.

### SLE mice display impaired ovarian reserve and reduced fertility

**Fig. 2:**
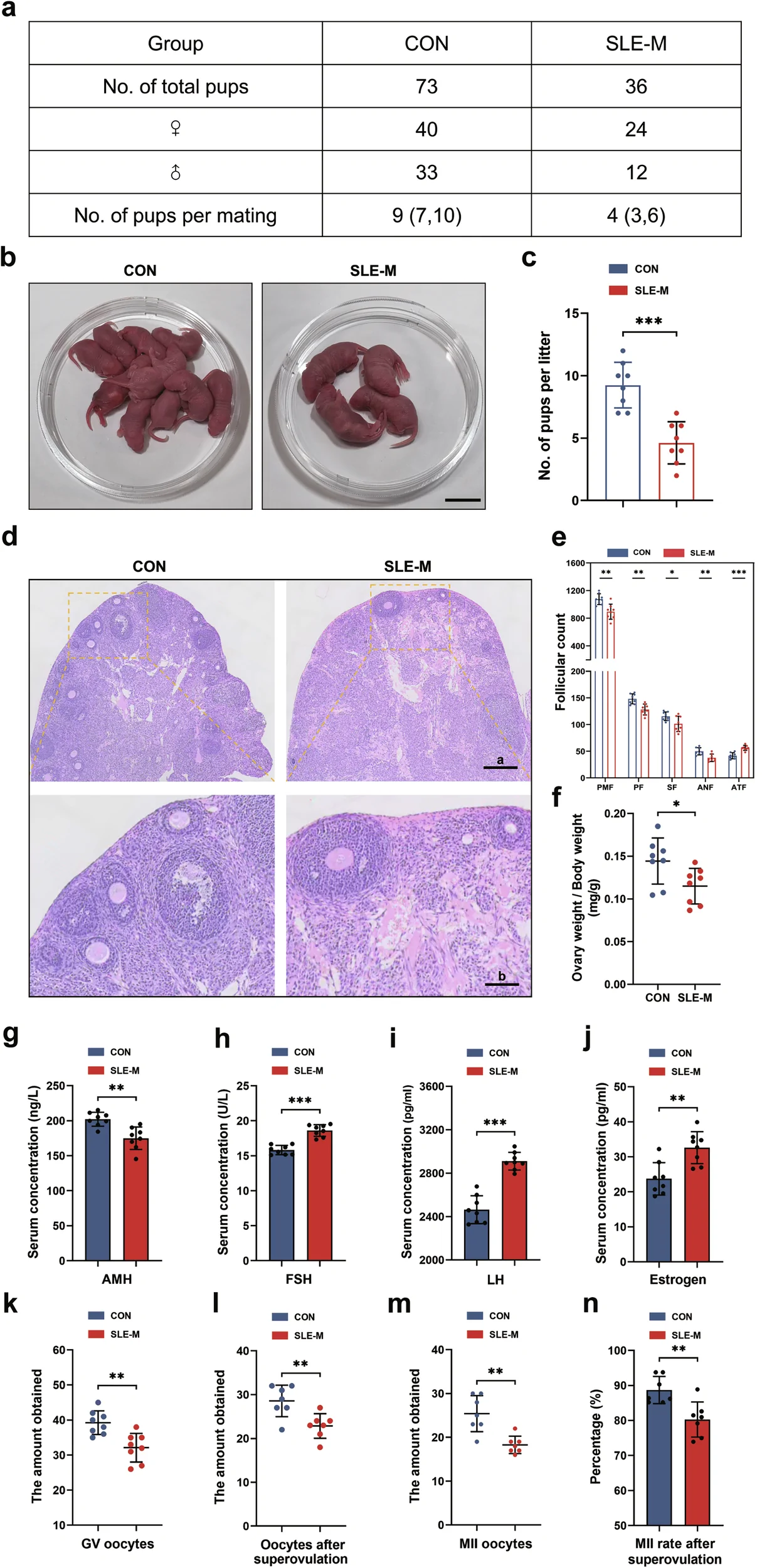
Impaired fertility occurred in the SLE-M mice. **a** Summary of total number of pups born in the CON and SLE-M groups. Data are presented as median with interquartile range (*n* = 8 litters per group). **b** Representative images of single litters from randomly selected pregnant mice in the CON and SLE-M groups. Scale bar, 2 cm. **c** Number of pups per litter in the CON and SLE-M mice (*n* = 8 litters per group). **d** Images of H&E staining of paraffin-embedded ovary sections at maximum cross from CON (*n* = 8) and SLE-M (*n* = 8) mice. Scale bar, a, 100 μm; **b** 30 μm. **e** Quantification of follicle numbers at different developmental stages (*n* = 8 mice per group). PMF, primordial follicle; PF, primary follicle; SF, secondary follicle; ANF, antral follicles; ATF, atretic follicles. **f** Ovary weight/body weight ratio in CON and SLE-M mice of 16 weeks (*n* = 8 mice per group). **g**–**j** Serum basal sex hormones of AMH, FSH, LH and E2 levels of CON mice and SLE-M mice at 16 weeks (*n* = 8 mice per group). **k** Number of obtained GV oocytes from 16-week-old CON and SLE-M mice without superovulation (*n* = 8 mice per group). **l**–**m** Number of obtained oocytes and mature oocytes after superovulation from 16-week CON and SLE-M mice (*n* = 7 mice per group); **n** The percentage of MII in oocytes from CON and SLE-M mice after superovulation (*n* = 7 mice per group). Data are presented as mean ± SD. Statistical analysis was performed using unpaired two-tailed Student’s *t*-test. *P < 0.05, **P < 0.01, ***P < 0.001.

Female SLE-M mice exhibited impaired reproductive performance, as reflected by a significant reduction in litter size compared with controls (Fig. 2a–c). Histological analysis revealed substantial disruption of ovarian architecture, including a marked reduction in follicles at all developmental stages and a concomitant increase in atretic follicles (Fig. 2d, e). Consistently, the ovarian-to-body weight ratio was significantly decreased in SLE-M mice (Fig. 2f). Hormonal profiling further supported diminished ovarian reserve, characterized by reduced AMH levels and increased FSH, LH, and E2 concentrations (Fig. 2g–j).

Functionally, both basal and stimulated oocyte yields were reduced in SLE-M mice. The number of GV oocytes retrieved without stimulation was decreased, and superovulation resulted in lower total oocyte numbers and reduced maturation rates (Fig. 2k–n).

### Oocyte developmental competence and embryo quality are compromised in SLE mice

**Fig. 3:**
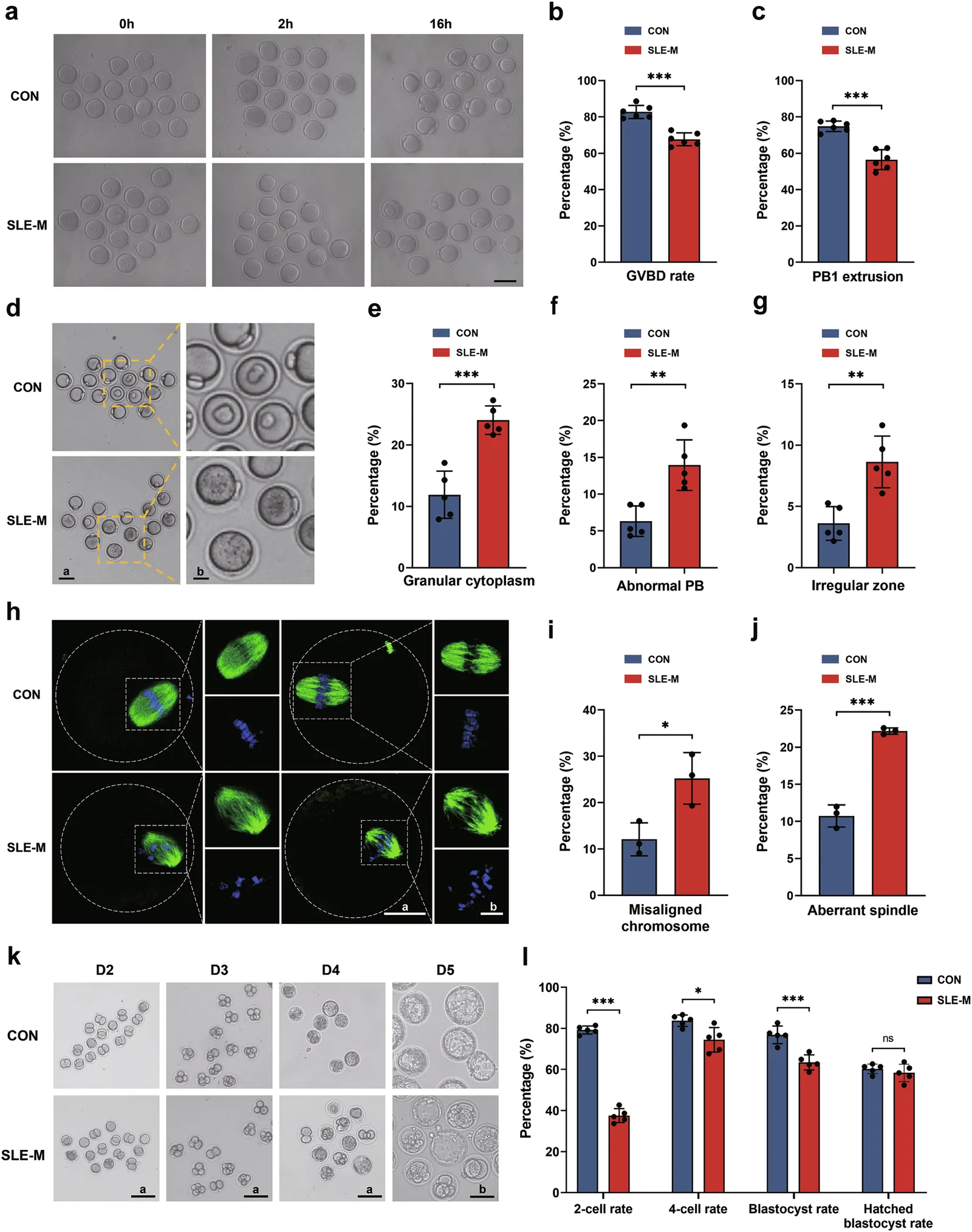
Developmental competence of oocytes was significantly decreased in the SLE-M mice. **a** Representative images of in vitro matured oocytes from CON and SLE-M group. Scale bar, 50 μm. **b**, **c** GVBD and PB1 extrusion rates (CON: *n* = 193 oocytes; SLE-M: *n* = 142 oocytes), derived from 6 independent experiments. **d** Representative morphology of MII oocytes from CON and SLE-M group. Scale bar, **a** 50 μm; **b** 20 μm. **e**–**g** Percentage of abnormal oocytes (granular cytoplasm, abnormal polar body, irregular zona pellucida) (CON: *n* = 195; SLE-M: *n* = 183 oocytes), derived from 6 independent experiments. **h** Typical image of the spindle and chromosome morphology in oocytes from CON and SLE-M mice. Scale bar, **a** 20 μm; **b** 10 μm. **i**–**j** The percentage of misaligned chromosomes and aberrant spindles in oocytes from CON (*n* = 74) and SLE-M mice (*n* = 81). **k** Representative embryo development after IVF in CON and SLE-M group. Scale bar, **a** 200 μm; **b** 100 μm. **I** The percentage of embryos at different developmental stages in CON and SLE-M mice after IVF (*n* = 5 independent IVF experiments). Data are presented as mean ± SD from at least three independent experiments. Statistical significance was determined using unpaired two-tailed Student’s *t*-test. *P < 0.05, **P < 0.01, ***P < 0.001.

Oocytes derived from SLE-M mice exhibited impaired developmental competence during in vitro maturation. Both GVBD at 2 h and first polar body (PB1) extrusion at 16 h were significantly reduced compared with controls (Fig. 3a–c). Morphological assessment revealed increased cytoplasmic abnormalities, including granular cytoplasm, abnormal polar body extrusion, and irregular zona pellucida (Fig. 3d–g). In addition, a higher proportion of oocytes displayed abnormal spindle organization and chromosome misalignment (Fig. 3h–j). Following in vitro fertilization, developmental progression was significantly impaired, with reduced rates of two-cell, four-cell, and blastocyst formation (Fig. 3k, l).

These findings were further supported by time-course analysis of meiotic progression, which revealed persistently reduced PB1 extrusion rates and increased MI arrest in SLE-M oocytes at multiple culture time points (Supplementary Fig. S1a–b). In addition, the proportions of abnormal oocyte morphology and spindle–chromosome defects were consistently elevated in SLE-M mice (Supplementary Fig. S1c–d). At the embryonic level, blastocysts derived from SLE-M oocytes exhibited reduced total cell numbers and ICM cell numbers (Supplementary Fig. S1e–g), accompanied by increased apoptosis as indicated by TUNEL staining (Supplementary Fig. S1h–j). These results further support impaired developmental competence of oocytes in SLE-M mice.

### Inflammation is associated with oocyte-quality decline and transcriptomic alterations

**Fig. 4:**
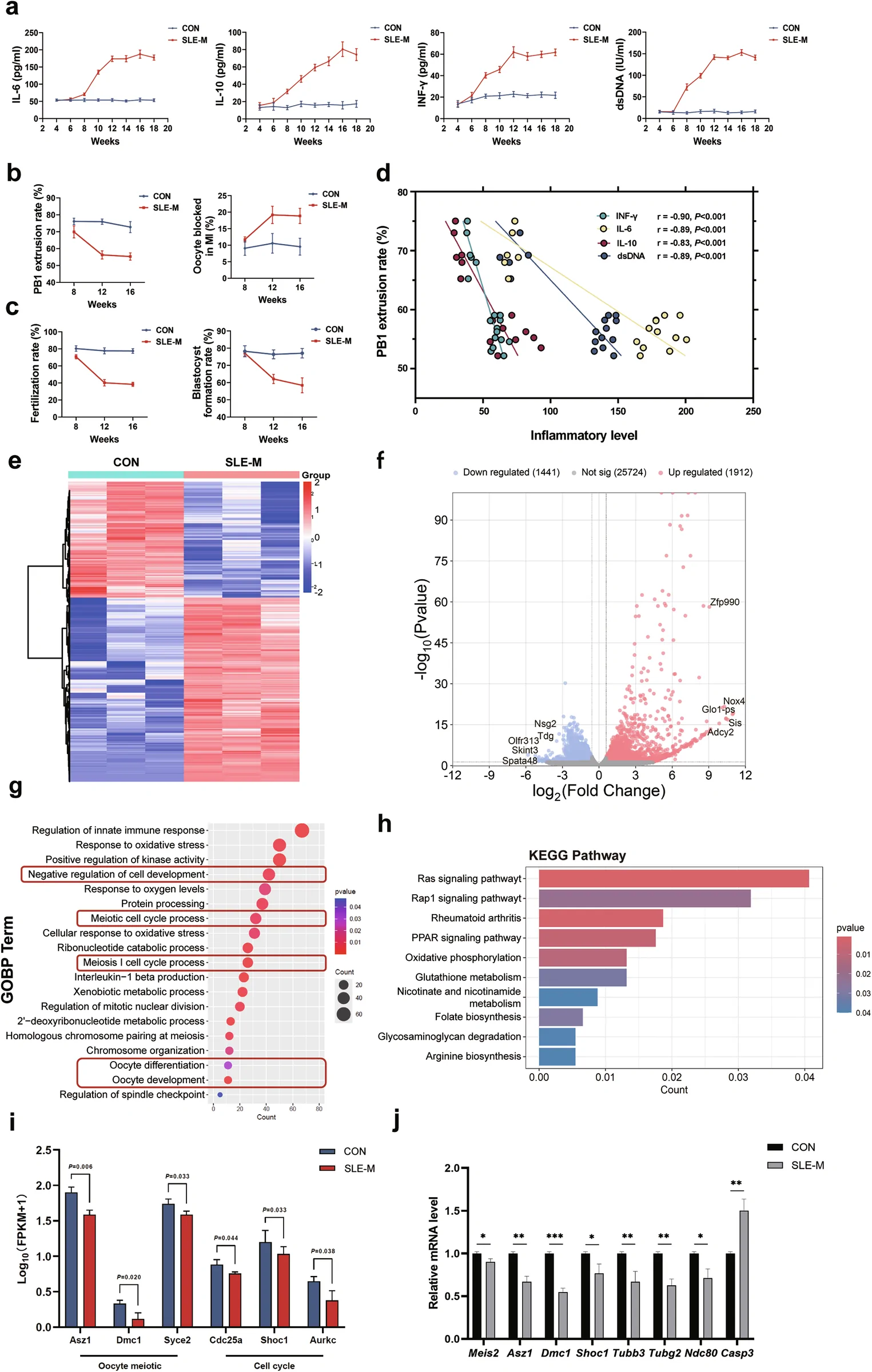
Oocyte development in SLE-M mice was negatively correlated with increased inflammatory status and related smart-seq profiling was exhibited. **a** Serum inflammatory markers (IL-6, IL-10, IFN-γ and dsDNA) across time points in CON and SLE-M group (*n* = 3 mice per group per time point). **b**, **c** PB1 extrusion, MI arrest, fertilization, and blastocyst rates in CON and SLE-M group (*n* = 3 independent experiments). **d** Correlation analysis between PB1 extrusion rate and serum inflammatory markers (IL-6, IL-10, IFN-γ, and anti-dsDNA). Each marker was analyzed separately, and values are presented in their respective units (pg/ml for cytokines and IU/ml for anti-dsDNA). Each dot represents one mouse from 8–18 weeks of age. **e**, **f** Heatmap and volcano plot of differentially expressed genes between CON and SLE-M group. **g** GO enrichment analysis. Red rectangles highlight representative GOBP terms associated with oocyte development and meiotic regulation. **h** KEGG enrichment analysis. **i**, **j** FPKM and RT-qPCR validation of selected genes involved in oocyte meiotic and cell cycle (*n* = 3 independent samples). Data are presented as mean ± SD. Statistical analysis was performed using unpaired two-tailed Student’s *t*-test. *P < 0.05, **P < 0.01, ***P < 0.001.

Longitudinal analysis revealed progressive increases in systemic inflammatory markers, including IL-6, IL-10, IFN-γ, and anti-dsDNA antibodies, from 4 to 18 weeks of age (Fig. 4a). This increase was accompanied by a gradual decline in oocyte developmental competence, including reduced PB1 extrusion, increased meiotic arrest, and decreased fertilization and blastocyst formation rates (Fig. 4b, c, Supplementary Fig. S2c). Correlation analyses demonstrated negative associations between PB1 extrusion rate and individual serum inflammatory markers (Fig. 4d).

Transcriptomic analysis of GV oocytes identified 3353 differentially expressed genes (1441 upregulated and 1912 downregulated; Fig. 4e, f). Gene Ontology enrichment revealed disruption of processes related to meiotic cell cycle and oocyte development (Fig. 4g), while KEGG analysis highlighted enrichment of oxidative stress–related pathways (Fig. 4h). RT-PCR validation confirmed dysregulation of genes involved in meiotic regulation (Asz1, Dmc1), cell cycle progression (Cdc25a), and cytoskeletal organization (Tubb3) (Fig. 4i, j). Consistently, several genes associated with oocyte development, spindle organization, and chromosome dynamics were downregulated in SLE-M oocytes, as shown by FPKM analysis (Supplementary Fig. S2d).

### HCQ treatment improves oocyte developmental outcomes in SLE mice

**Fig. 5:**
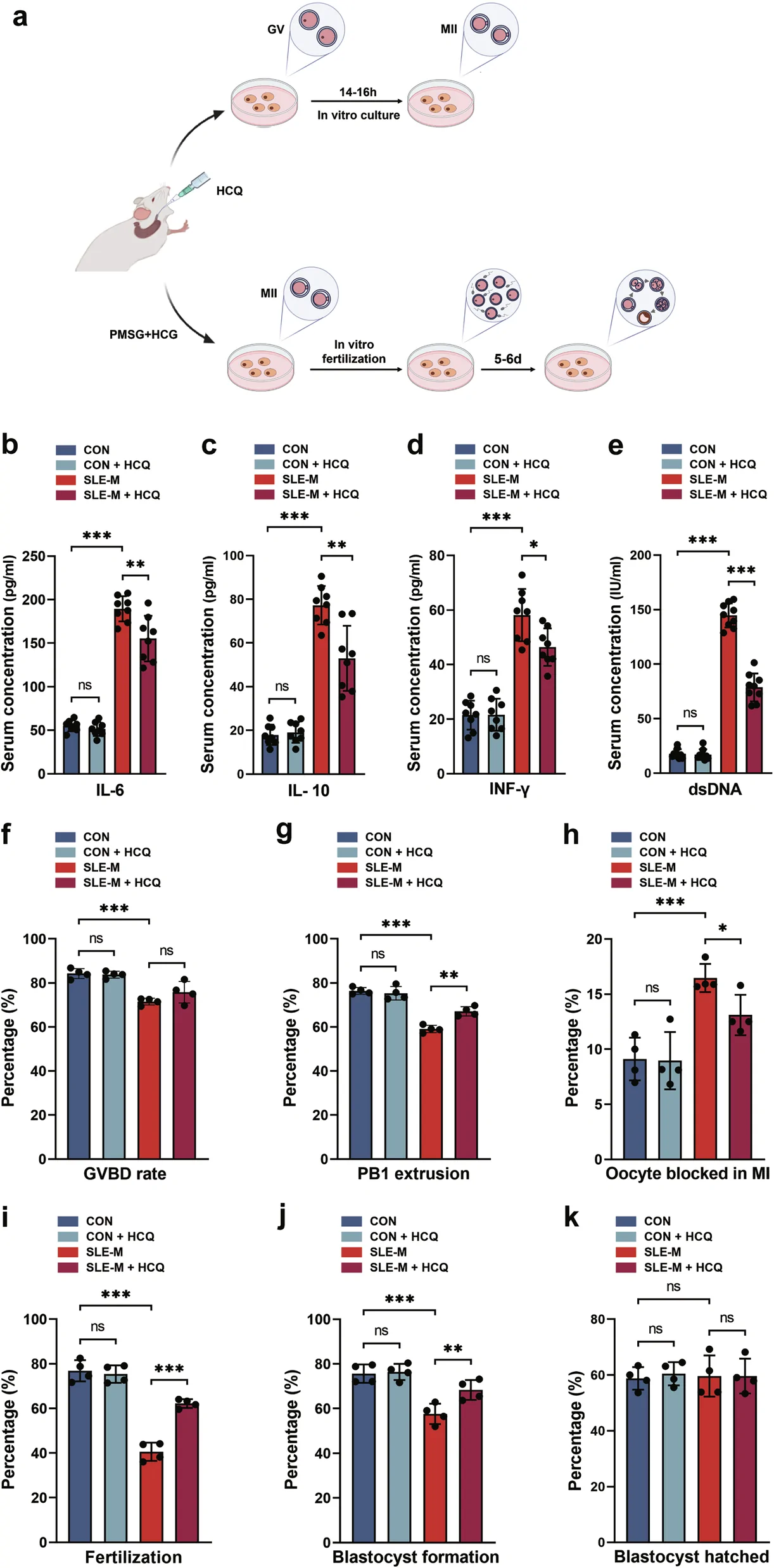
HCQ treatment reduces systemic inflammation and improves oocyte developmental outcomes in SLE-M mice. **a** Diagram of the experimental design after hydroxychloroquine gavage treatment. **b**–**e** The level of serum inflammatory markers (IL-6, IL-10, IFN-γ, and anti-dsDNA antibody) in CON and SLE-M group (*n* = 8 mice per group). The percentage of oocyte development including GVBD (**f**), PB1 extrusion (**g**) and MI blocking (**h**) in oocytes from CON (*n* = 193), CON + HCQ (*n* = 185), SLE-M (*n* = 207), and SLE-M + HCQ (*n* = 177) mice. **i**–**k** The percentage of embryo development including fertilization (**i**), blastocyst formation (**j**) and blastocyst hatched (**k**) in CON (*n* = 117), CON + HCQ (*n* = 118), SLE-M (*n* = 112), and SLE-M + HCQ (*n* = 108) group. Data are presented as mean ± SD. Statistical analysis was performed using one-way ANOVA followed by Tukey’s multiple comparison test. *P < 0.05, **P < 0.01, ***P < 0.001.

Treatment with hydroxychloroquine (HCQ) reduced systemic inflammation, as indicated by decreased serum levels of IL-6, IL-10, IFN-γ, and anti-dsDNA antibodies (Fig. 5a–e).

Compared with untreated SLE-M mice, HCQ-treated mice exhibited improved oocyte developmental outcomes, including increased PB1 extrusion rates, reduced meiotic arrest, and higher fertilization and blastocyst formation rates (Fig. 5f–k). These findings indicate that attenuation of inflammation is associated with improved oocyte and embryo development in SLE mice.

### Transcriptomic and biochemical analyses indicate enhanced oxidative stress in SLE mice

**Fig. 6:**
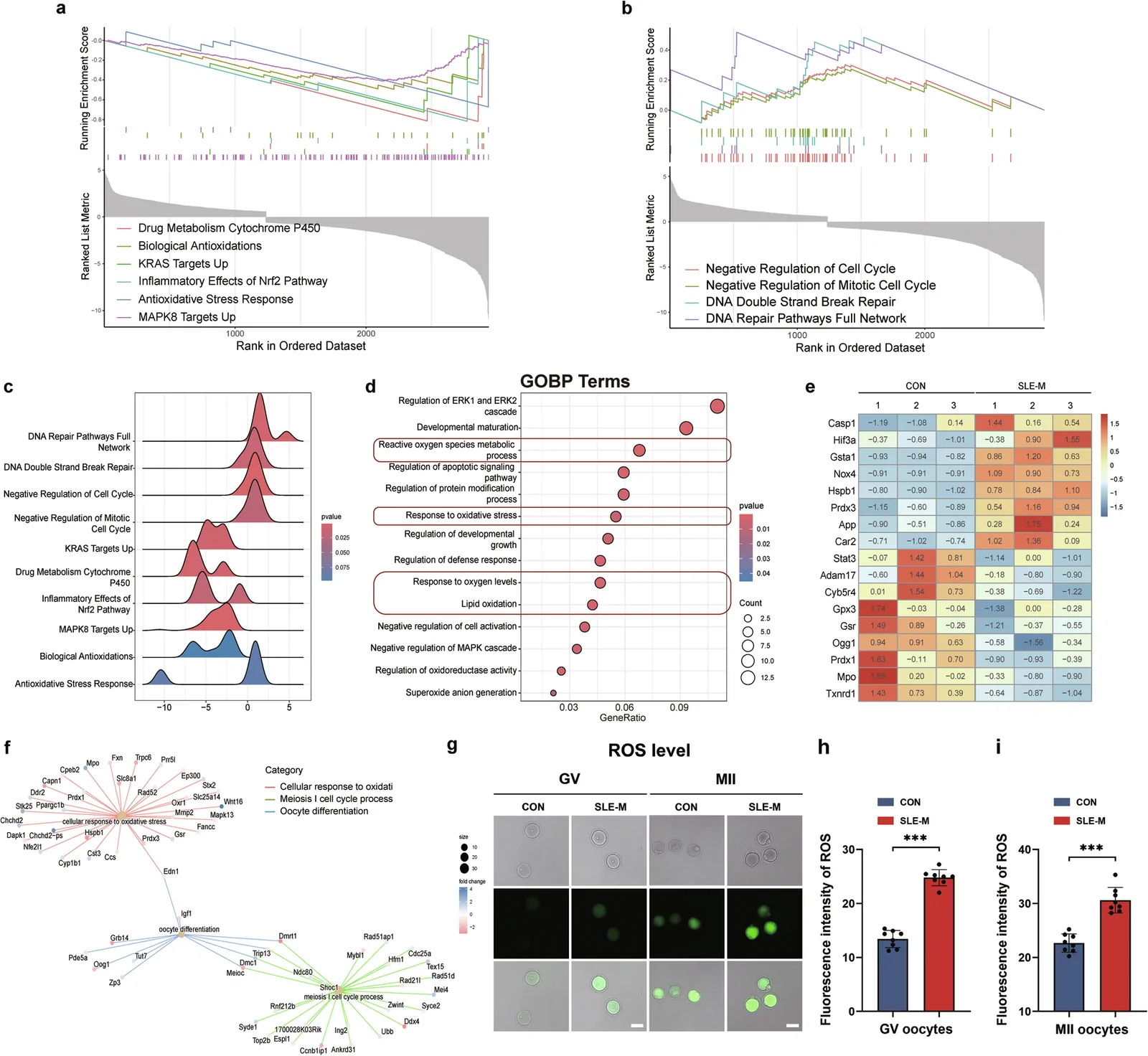
The level of oxidative stress in oocytes from SLE-M mice was distinctly increased and was closely related to the level of inflammation. **a** Downregulated pathways and corresponding enrichment score in GSEA analysis. **b** Upregulated pathways and corresponding enrichment score in GSEA analysis. **c** Enriched pathways presented in ridgeplot for GSEA analyses. **d**, **e** GO analysis and heatmap of oxidative stress-related genes. **f** The cnetplot of differential genes in oocytes from CON and SLE-M group, involved in meiosis cell cycle process, oocyte differentiation and cellular response to oxidative stress. **g** Representative ROS staining of GV and MII oocytes from CON and SLE-M group. Scale bars, 50μm. **h**, **i** Quantification of ROS fluorescence intensity in GV and MII oocytes from CON (*n* = 8) and SLE-M (*n* = 8) group. Data are presented as mean ± SD. Statistical analysis was performed using unpaired two-tailed Student’s *t*-test. *P < 0.05, **P < 0.01, ***P < 0.001.

Gene set enrichment analysis revealed downregulation of antioxidant pathways and upregulation of DNA damage–related pathways in SLE-M oocytes (Fig. 6a–c). Gene Ontology analysis further indicated enrichment of oxidative stress–related processes (Fig. 6d), and clustering analysis showed clear separation between SLE-M and control groups based on oxidative stress–related genes (Fig. 6e). Network analysis further illustrated the interaction of differentially expressed genes involved in meiotic cell cycle regulation, oocyte differentiation, and cellular response to oxidative stress (Fig. 6f), supporting coordinated alterations in pathways associated with oocyte function and redox homeostasis.

Consistently, immunofluorescence analysis demonstrated increased ROS levels in both GV and MII oocytes from SLE-M mice (Fig. 6g–i). In line with these findings, systemic oxidative stress markers were altered in SLE-M mice, as evidenced by decreased GSH-PX and SOD levels and increased MDA levels (Supplementary Fig. S3a–c). In addition, expression of ROS-related genes was dysregulated (Supplementary Fig. S3d). Time-course analysis further revealed sustained elevation of ROS levels in oocytes from SLE-M mice (Supplementary Fig. S3e, f), supporting the presence of persistent oxidative stress. To further assess the functional relevance of oxidative stress, we examined the effects of HCQ on oocyte redox status and mitochondrial function. HCQ treatment significantly reduced intracellular ROS accumulation, modulated the expression of redox-related genes, and improved mitochondrial distribution patterns and membrane potential (Supplementary Fig. S4a–g).

### Melatonin alleviates oxidative stress-induced mitochondrial dysfunction in vivo and in vitro

**Fig. 7:**
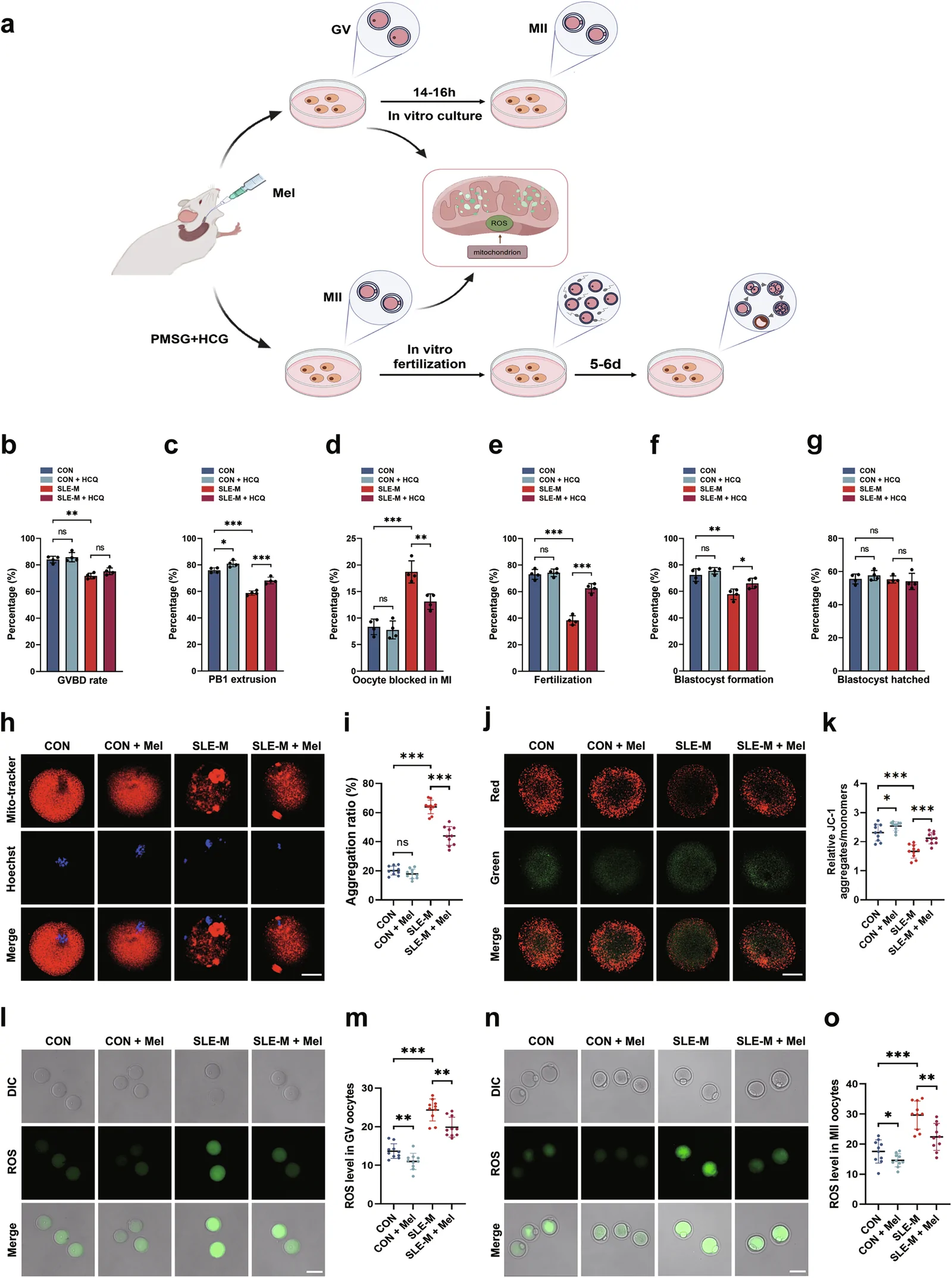
Melatonin alleviates oxidative stress-associated mitochondrial abnormalities and improves oocyte developmental outcomes in SLE-M mice in vivo. **a** Schematic diagram of the experimental design for in vivo melatonin treatment. **b**–**d** The percentage of oocyte development including GVBD (**b**), PB1 extrusion (**c**) and MI blocking (**d**) in oocytes from CON (*n* = 168), CON+Mel (*n* = 159), SLE-M (*n* = 194), and SLE-M+Mel (*n* = 156) group. The percentage of embryo development including fertilization (**e**), blastocyst formation (**f**) and blastocyst hatched (**g**) in CON (*n* = 122), CON+Mel (*n* = 127), SLE-M (*n* = 111), and SLE-M+ Mel (*n* = 97) group. **h**, **i** Representative images of mitochondrial distribution in oocytes from CON, CON+Mel, SLE-M, and SLE-M+Mel group, and quantification of the mitochondrial aggregation ratio. The aggregation ratio was calculated as the percentage of oocytes showing evident mitochondrial aggregation in each independent experiment (*n* = 10 independent experiments). Scale bar, 20μm. **j**, **k** Representative images of MMP stained in CON, CON+Mel, SLE-M, and SLE-M+Mel group, and corresponding analysis (JC-1 red/green ratio; *n* = 10 oocytes per group). Scale bar, 20 μm. **l**–**o** Representative images of ROS in GV and MII oocytes from CON, CON+Mel, SLE-M, and SLE-M+Mel group, and quantitative fluorescence intensity (*n* = 10 oocytes per group). Scale bar, 50μm. Data are presented as mean ± SD. Statistical analysis was performed using one-way ANOVA followed by Tukey’s multiple comparison test. *P < 0.05, **P < 0.01, ***P < 0.001.

To further investigate whether oxidative stress contributes to oocyte defects in SLE, we evaluated the effects of melatonin treatment on oocyte developmental competence. In vivo administration of melatonin significantly increased polar body extrusion rates and reduced meiotic arrest in SLE-M oocytes (Fig. 7a–g). Similarly, in vitro supplementation with melatonin enhanced GVBD and PB1 extrusion rates, as well as subsequent embryo developmental potential (Supplementary Fig. S5a-g), indicating an improvement in both nuclear maturation and developmental competence.

Given the established association of mitochondrial function with oocyte competence [33, 34], and the close relationship between ROS and mitochondrial homeostasis [35], we next assessed mitochondrial organization and function following melatonin treatment. As shown in Fig. 7h–k and Supplementary Fig. S5h-k, melatonin treatment restored mitochondrial distribution patterns and improved mitochondrial membrane potential (ΔΨm), as evidenced by JC-1 staining. Along with these changes, a significant reduction in intracellular ROS levels was observed in both GV and MII oocytes following melatonin treatment (Fig. 7l–o and Supplementary Fig. S5l-m), suggesting an attenuation of oxidative stress. Furthermore, melatonin treatment improved intracellular redox balance, as indicated by increased NADPH/NADP⁺ and GSH/GSSG ratios, together with partial normalization of redox-related gene expression (Supplementary Fig. S5n-p). These findings collectively indicate that melatonin alleviates oxidative stress–associated mitochondrial dysfunction and improves oocyte developmental competence in SLE.

### Ferroptosis-associated alterations are observed in SLE oocytes and are attenuated by melatonin

**Fig. 8:**
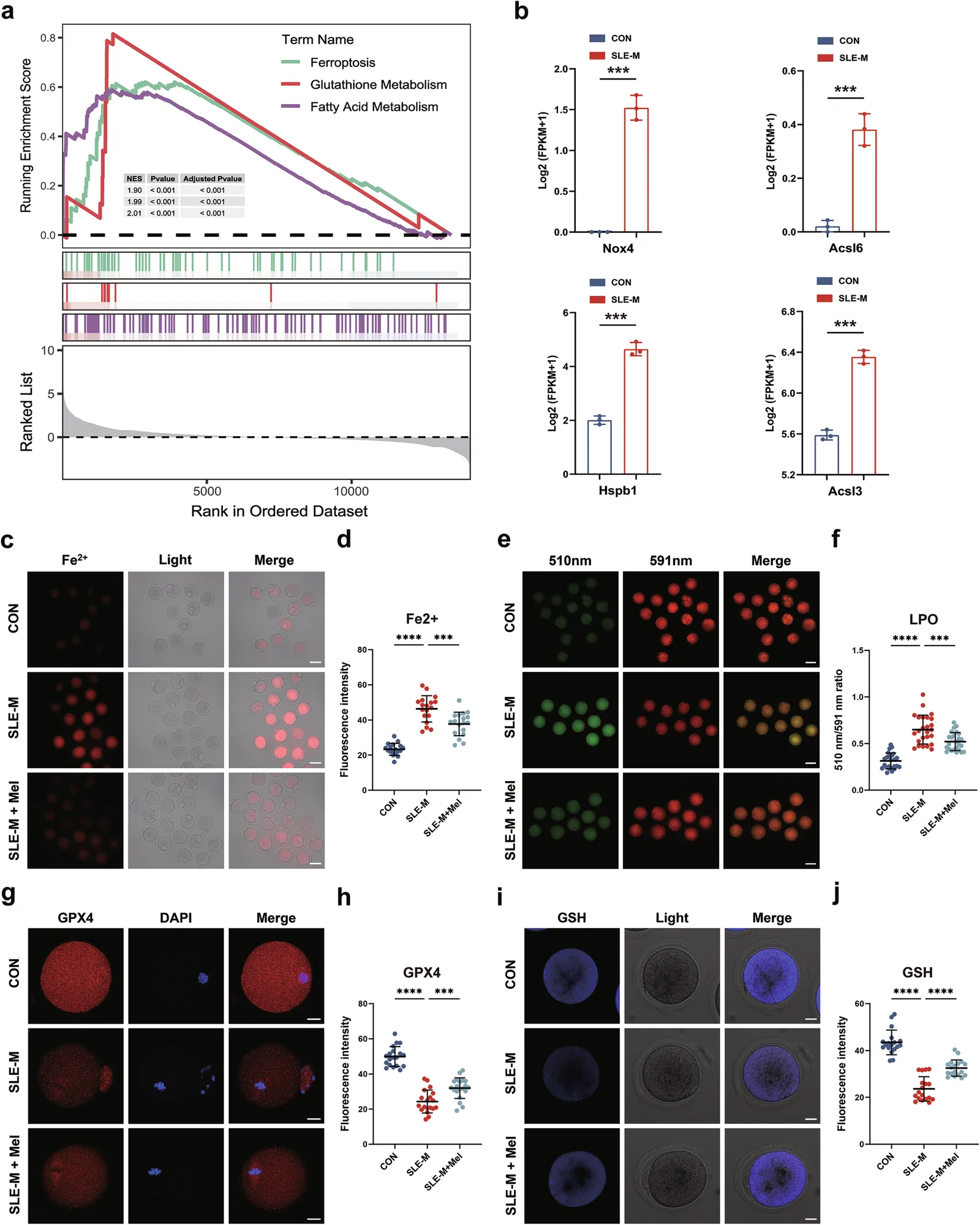
Ferroptosis-associated alterations are observed in SLE-M oocytes and are attenuated by melatonin. **a** GSEA plots showing upregulation of ferroptosis-associated pathways in SLE-M oocytes. **b** Heatmap of normalized log2(FPKM + 1) values for representative ferroptosis-related genes in the CON and SLE-M groups. **c** Representative fluorescence images of FerroOrange staining for labile Fe²⁺. Scale bar, 50 μm. **d** Quantification of FerroOrange fluorescence intensity in the CON, SLE-M, and SLE-M+Mel groups (*n* = 18 oocytes per group). **e** Representative fluorescence images of BODIPY 581/591 C11 staining for lipid peroxidation. Scale bar, 50 μm. **f** Quantification of BODIPY C11 fluorescence intensity in the CON, SLE-M, and SLE-M+Mel groups (*n* = 25 oocytes per group). **g** Representative immunofluorescence images of GPX4 expression. Scale bar, 10 μm. **h** Quantification of GPX4 fluorescence intensity in the CON (*n* = 19), SLE-M (*n* = 19), and SLE-M+Mel (*n* = 20) groups. **i** Representative images of cellular GSH staining. Scale bar, 10 μm. **j** Quantification of GSH fluorescence intensity in the CON, SLE-M, and SLE-M+Mel groups (*n* = 18 oocytes per group). Data are presented as mean ± SD. Statistical analysis was performed using one-way ANOVA followed by Tukey’s multiple comparison test. *P < 0.05, **P < 0.01, ***P < 0.001.

To determine whether ferroptosis-related alterations are present in SLE oocytes, we first performed transcriptomic analyses. Gene set enrichment analysis revealed activation of ferroptosis-associated pathways in SLE-M oocytes (Fig. 8a), and distinct expression patterns of ferroptosis-related genes were observed compared with controls (Fig. 8b).

We next examined key biochemical features associated with ferroptosis. As shown in Fig. 8c–f, SLE-M oocytes exhibited significantly elevated intracellular Fe²⁺ levels and increased lipid peroxidation. In parallel, GPX4 expression was decreased and intracellular glutathione levels were reduced (Fig. 8g–j), indicating disruption of cellular redox homeostasis. Notably, melatonin treatment attenuated these alterations, as evidenced by reduced lipid peroxidation and Fe²⁺ accumulation, together with restoration of glutathione levels and GPX4 expression (Fig. 8c–j). These changes were observed in parallel with the reduction of oxidative stress and improvement of mitochondrial function described above.

Collectively, these findings indicate that ferroptosis-associated alterations are present in SLE oocytes and can be attenuated by melatonin in parallel with improvements in redox balance and mitochondrial function.

### Ferroptosis inhibition with ferrostatin-1 partially rescues oocyte defects in SLE mice

**Fig. 9:**
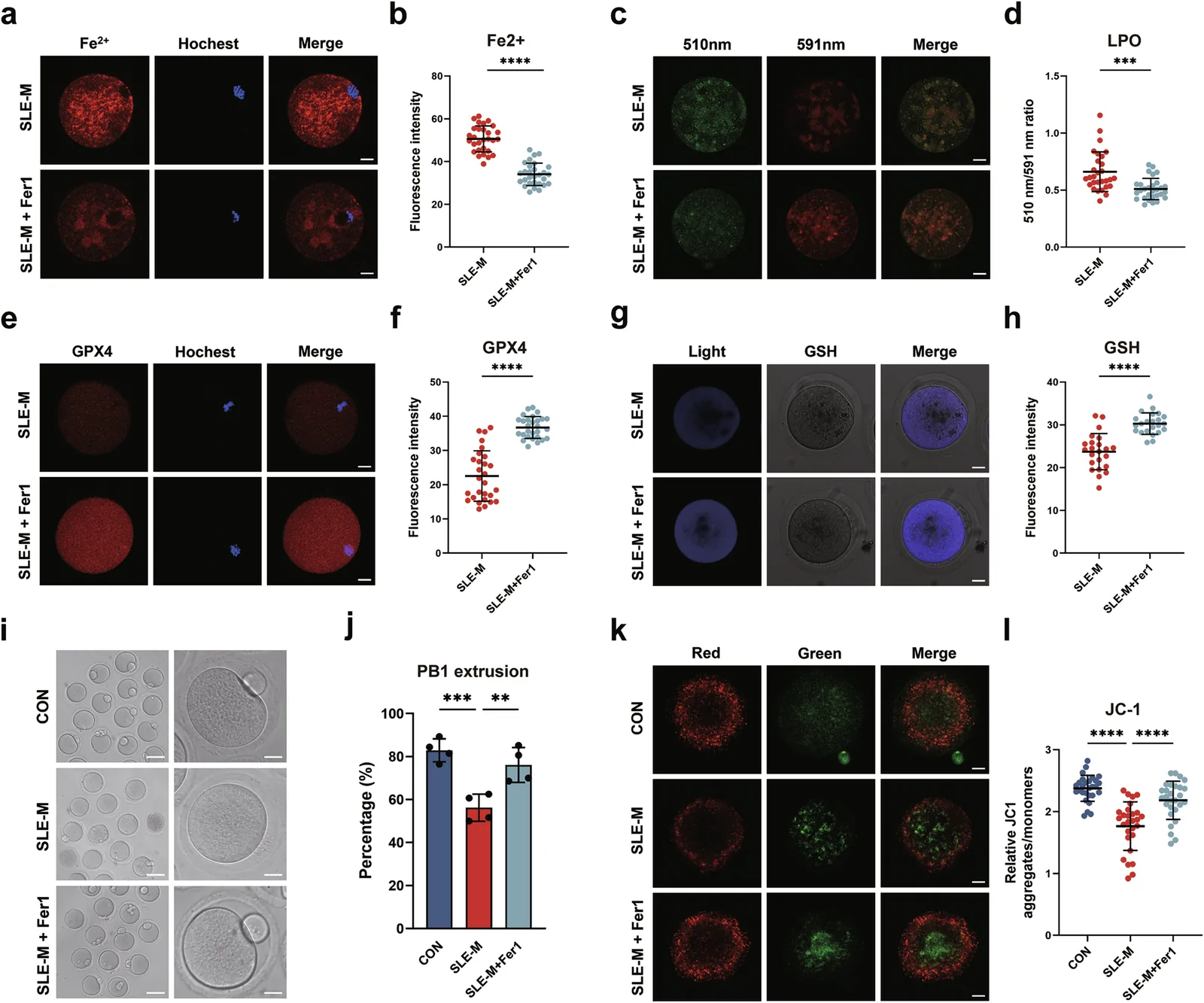
Ferroptosis inhibition with ferrostatin-1 partially rescues oocyte defects in vitro. **a**, **b** Representative fluorescence images and quantification of intracellular Fe²⁺ levels in the SLE-M (*n* = 29) and SLE-M+Fer-1 (*n* = 29) groups. Scale bar, 10 μm. **c**, **d** Representative fluorescence images and quantification of lipid peroxidation in the SLE-M (*n* = 29) and SLE-M+Fer-1 (*n* = 29) groups. Scale bar, 50 μm. **e**, **f** Representative fluorescence images and quantification of GPX4 expression in the SLE-M (*n* = 28) and SLE-M+Fer-1 (*n* = 28) groups. Scale bar, 50 μm. **g**–**h** Representative fluorescence images and quantification of GSH levels in the SLE-M and SLE-M+Fer-1groups (*n* = 23 oocytes per group). Scale bar, 50 μm. **i** Representative images of PB1 extrusion in oocytes from the CON, SLE-M, and SLE-M+Fer-1 groups. Low-magnification (left) and high-magnification (right) images are shown for each group. Scale bars: left, 50 μm; right, 10 μm. **j** Quantification of PB1 extrusion rates in the CON (*n* = 121 oocytes), SLE-M (*n* = 118 oocytes), and SLE-M+Fer-1 (*n* = 124 oocytes) groups, derived from 4 independent experiments. **k**, **l** Representative fluorescence images and quantification of mitochondrial membrane potential (JC-1 red/green fluorescence ratio) in the CON (*n* = 28), SLE-M (*n* = 28), and SLE-M+Fer-1 (*n* = 28) groups. Scale bar, 10 μm. Data are presented as mean ± SD. Statistical analysis was performed using one-way ANOVA followed by Tukey’s multiple comparison test. *P < 0.05, **P < 0.01, ***P < 0.001.

To further evaluate whether ferroptosis is functionally involved in SLE-associated oocyte impairment, GV oocytes were cultured in vitro with the ferroptosis inhibitor ferrostatin-1 (Fer-1). Inhibition of ferroptosis was first assessed by examining ferroptosis-associated biochemical features. Fer-1 treatment markedly reduced intracellular Fe²⁺ levels and lipid peroxidation in SLE-M oocytes (Fig. 9a–d). Consistently, GPX4 expression was increased following Fer-1 treatment (Fig. 9e, f), and intracellular GSH levels were also elevated (Fig. 9g, h).

In parallel, Fer-1 treatment partially restored oocyte meiotic maturation capacity, as reflected by an increased PB1 extrusion rate compared with untreated SLE-M oocytes (Fig. 9i, j).

In addition, mitochondrial function was improved following ferroptosis inhibition, as indicated by an increased mitochondrial membrane potential (ΔΨm) measured by JC-1 staining (Fig. 9k, l). Together, these results suggest that inhibition of ferroptosis partially rescues oocyte defects in SLE-M mice.

## Discussion

Our previous study identified a genetic link between SLE and primary ovarian failure (POF) [12], and clinical cohort analyses further demonstrated impaired ovarian reserve and oocyte quality in women with SLE [13]. In the present study, our data suggest that SLE-associated inflammation is accompanied by impaired female fertility in mice, as reflected by altered hormone profiles, disrupted follicular development, and reduced oocyte developmental competence. In addition to systemic inflammatory activation, increased CD3- and CD68-positive cell infiltration in SLE ovaries supports the presence of a locally altered ovarian inflammatory microenvironment. Mechanistically, our findings indicate that inflammation is associated with oxidative stress, mitochondrial dysfunction, and ferroptosis-related alterations in oocytes. Notably, HCQ and melatonin alleviated oxidative stress–related abnormalities and improved oocyte developmental outcomes, while ferrostatin-1 (Fer-1) provided functional evidence supporting the involvement of ferroptosis in this process. From multiple perspectives, our findings are broadly consistent with previous clinical observations of impaired ovarian reserve in women with SLE [9, 36], as well as reduced family size in this population [10]. However, not all clinical outcomes fully align with the present experimental results. In our previous IVF cohort, early embryologic parameters such as maturation and fertilization rates were not significantly altered [13], whereas more pronounced defects were observed in the current mouse model. This discrepancy may be partly explained by differences in disease status, species differences and experimental context. Patients undergoing assisted reproduction are typically managed under conditions of controlled or low disease activity and often receive ongoing treatment, which may partially suppress systemic inflammation. In contrast, the MRL/lpr model represents sustained and untreated autoimmune activation, likely exposing oocytes to prolonged inflammatory stress and thereby revealing earlier and more severe reproductive defects. In addition, reduced family size in women with SLE may not solely reflect intrinsic reproductive dysfunction, but may also be influenced by psychological burden, reproductive concerns, and medication exposure [37]. Together, these observations suggest that reproductive outcomes in SLE reflect both biological impairment and external influences.

Our data further support that deterioration of oocyte competence is a central component of SLE-associated reproductive impairment. In SLE mice, this was characterized by reduced meiotic maturation, spindle–chromosome abnormalities, and impaired embryonic development. These findings are partly consistent with our previous clinical observations of reduced blastocyst developmental potential in women with SLE [13], while extending them by revealing earlier-stage defects under sustained inflammatory conditions. Given the central role of mitochondria in regulating oocyte quality, the observed mitochondrial abnormalities further support a subcellular basis for lupus-associated oocyte injury.

Inflammation is a hallmark feature of SLE and appears to be closely linked to oocyte dysfunction [38, 39]. In the present study, inflammatory status was negatively correlated with oocyte maturation across different ages, and HCQ-treated SLE mice exhibited improved oocyte developmental outcomes compared with untreated SLE mice. Together with the evidence of ovarian immune-cell infiltration, these findings support the notion that both systemic and local inflammatory disturbance contribute to impaired oocyte competence in SLE. Accordingly, anti-inflammatory strategies may partially mitigate immune-associated reproductive dysfunction.

Inflammation and oxidative stress form a self-amplifying loop in autoimmune diseases such as SLE [40–43]. Consistent with this, SLE mice exhibited increased oxidative burden and reduced antioxidant capacity, accompanied by transcriptomic signatures related to oxidative stress. Excessive ROS has been implicated in multiple reproductive disorders, including preeclampsia [44], birth defects [45], miscarriage [46], infertility [47]. Moreover, oxidative stress disrupts mitochondrial function and damages key subcellular structures in oocytes [48, 49]. Our findings extend these observations by linking inflammation-associated oxidative stress to mitochondrial dysfunction and impaired oocyte competence in SLE.

Beyond generalized oxidative stress, our study provides converging evidence that ferroptosis is involved in SLE-associated oocyte damage [20]. Ferroptosis, an iron-dependent form of regulated cell death driven by lipid peroxidation, has recently emerged as an important mechanism in reproductive disorders [50], including PCOS [51], premature ovarian insufficiency [52], endometriosis [53], and pregnancy-related complications [54]. In our model, SLE oocytes exhibited multiple ferroptosis-associated features, including iron accumulation, glutathione depletion, increased lipid peroxidation, and reduced GPX4 expression [55]. Additionally, Melatonin attenuated these alterations in parallel with reduced oxidative stress and improved developmental outcomes, suggesting that oxidative stress may act as an upstream driver of ferroptosis-associated alterations.

Importantly, the Fer-1 rescue experiments strengthen the evidence linking ferroptosis to oocyte damage in SLE. Pharmacological inhibition of ferroptosis partially restored meiotic maturation and ameliorated ferroptosis-associated alterations, redox imbalance, and mitochondrial dysfunction. These findings provide functional evidence supporting the involvement of ferroptosis in SLE-associated oocyte damage.

## Conclusion

In summary, our study suggests that SLE-associated inflammation is associated with oxidative stress, mitochondrial dysfunction, and ferroptosis-related alterations, which contribute to impaired oocyte quality. The observed effects of anti-inflammatory, antioxidant, and ferroptosis-targeting interventions provide supportive evidence for the involvement of these pathways. Further studies are required to validate these findings in human oocytes and to explore potential strategies for fertility preservation in women with SLE.

## Supplementary information

Supplementary Information

## Data Availability

The experimental data necessary to support the conclusions of this study are provided in the main manuscript and/or the Supplementary Materials. Raw sequencing data have been deposited in the Genome Sequence Archive (GSA) at the BIG Data Center, Beijing Institute of Genomics (BIG), Chinese Academy of Sciences (https://bigd.big.ac.cn/), under accession number CRA030414.
