# Supplementary material for: Inflammation impairs fertility and oocyte quality in systemic lupus erythematosus by triggering oxidative stress-mediated ferroptosis
Source: Cell Death Dis. 2026 Jun 30;17(1):807. doi: 10.1038/s41419-026-09028-x (PMC13586256; doi:10.1038/s41419-026-09028-x)
Supplement: Supplementary file 1 [file 41419_2026_9028_MOESM1_ESM.docx]

**Supplementary Materials for**

**Inflammation impairs fertility and oocyte quality in systemic lupus erythematosus by triggering oxidative stress-mediated ferroptosis**

**Authors and affiliations:**

Ruolin Mao^1,2^, Juepu Zhou^1,2^, Xiangfei Wang^1,2^, Rui Long^1,2^, Meng Wang^1,2^, Hong Chen^1,2^, Youzhu Li ^3,4^, Nan Xiao^5,6^, Qingsong Xi^1,2*^ Lei Jin1^1,2^*, Lixia Zhu^1,2^*

1 Reproductive Medicine Center, Tongji Hospital, Tongji Medical College, Huazhong University of Science and Technology, Wuhan, China

2 National Clinical Research Center for Obstetrics and Gynecology, Tongji Hospital, Tongji Medical College, Huazhong University of Science and Technology, Wuhan, China.

3 Department of Reproductive Medicine, The First Affiliated Hospital of Xiamen University, School of Medicine, Xiamen University, Xiamen, China.

4 State Key Laboratory of Animal Biotech Breeding, Key Laboratory of Animal Genetics, Breeding and Reproduction of the Ministry of Agriculture, Frontiers Science Center for Molecular Design Breeding (MOE), College of Animal Science and Technology, China Agricultural University, Beijing, China.

5 Department of Center for Reproductive Medicine, Tianjin Central Hospital of Obstetrics and Gynecology, Tianjin, China.

6 Nankai University Affiliated Maternity Hospital, Tianjin Key Laboratory of Human Development and Reproductive Regulation, Tianjin, 300100, China.

**Supplementary Fig. S1. Developmental competence of oocytes was significantly decreased in the SLE-M mice.**

(a) The percentage of PB1 extrusion at different cultured time point from CON (n = 193) and SLE-M mice (n = 142). (b) The percentage of MI blocking at different cultured time point from CON (n = 193) and SLE-M mice (n = 142). (c) The ratio of three abnormal types of oocyte morphology in CON (n = 195) and SLE-M group (n = 183). (d) The ratio of abnormal spindle and chromosome in CON (n = 195) and SLE-M group (n = 183). (e) Representative confocal microscopy images of dyed ICM and total cell of blastocysts from CON and SLE-M mice. Scale bar, 50μm. (f-g) The comparison of total cell numbers of blastocyst and cell number of ICM between two groups (n=14 per group). Each dot represents one blastocyst.

(h) Representative confocal microscopy images of TUNEL assay of blastocysts in CON and SLE-M mice. Scale bars, 50μm. (i-j) The comparison of number and proportion of apoptosis-positive cells (red) between the two groups (n=9 per group).

Data are presented as mean ± SD. Statistical analysis was performed using unpaired two-tailed Student’s t-test. *P < 0.05, **P < 0.01, ***P < 0.001.

**Supplementary Fig. S2. Additional inflammatory and developmental alterations in SLE-M mice.**
(a) Representative immunohistochemical staining of CD68 in ovarian sections from CON and SLE-M group. Scale bar, 30 μm. (b) Quantification of CD68 staining intensity in ovarian tissues (n = 5 mice per group). (c) Rate of hatched blastocysts at different time points in CON and SLE-M mice (n = 3 mice per group). (d) Expression levels (FPKM) of representative genes related to oocyte development, spindle formation, and chromosome organization in CON and SLE-M oocytes, derived from RNA-seq analysis. Data are presented as mean ± SD. Statistical analysis was performed using unpaired two-tailed Student’s t-test.

**Supplementary Fig. S3.** **Serum oxidative stress markers, ROS analysis, and gene expression in SLE-M oocytes.**

(a-c) Comparison of the serum concentrations of GSH-PX, MDA and SOD in CON and SLE-M mice (n = 6 mice per group). (d) Relative mRNA expression levels of ROS-related genes in CON and SLE-M mice (n = 3 per group). (e-f) Representative confocal images of ROS staining in oocytes from CON and SLE-M mice at different weeks (n = 3 independent experiments). Scale bars, 50 μm. Data are presented as mean ± SD. Statistical analysis was performed using unpaired two-tailed Student’s t-test. *P < 0.05, **P < 0.01, ***P < 0.001.

**Supplementary Fig. S4. Hydroxychloroquine anti-inflammatory treatment reduced the level of ROS and subsequent mitochondrial imbalances in oocytes, thereby improving oocyte quality.**

(a-b) Representative confocal images of ROS in oocytes after hydroxychloroquine treatment, and corresponding quantitative analysis of ROS signal (n = 10 oocytes per group). Scale bar, 50μm. (c) Relative mRNA expression levels of ROS-related genes in MII oocytes after hydroxychloroquine treatment (n = 3). (d-e) Representative images of mitochondrial distribution in oocytes from CON, CON+HCQ, SLE-M, and SLE-M+HCQ group, and quantification of the mitochondrial aggregation ratio (n = 10 independent experiments). Scale bar, 20μm. (f-g) Representative images of MMP in oocytes from CON, CON+HCQ, SLE-M, and SLE-M+HCQ group, and corresponding quantitative analysis (n = 10 oocytes per group). Scale bar, 20μm. Data are presented as mean ± SD. Statistical analysis was performed using one-way ANOVA followed by Tukey’s multiple comparison test. *P < 0.05, **P < 0.01, **P < 0.001.

**Supplementary Fig. S5. Melatonin alleviated ROS-mediated mitochondrial imbalances of oocytes and improves oocyte development in SLE-M mice in vitro.**

(a) Diagram of the experimental design after in vitro melatonin treatment. (b-d) The percentage of oocyte development including GVBD, PB1 extrusion and MI blocking in oocytes from CON (n = 208), CON+ Mel (n = 202), SLE-M (n = 194), and SLE-M+ Mel (n = 177) group. (e-f) The percentage of embryo development including fertilization, blastocyst formation and blastocyst hatched in CON (n = 116), CON+ Mel (n = 125), SLE-M (n = 104), and SLE-M+ Mel (n = 103) group. (h-i) Representative images of mitochondrial distribution in CON, CON+Mel, SLE-M, and SLE-M+Mel group, and quantification of the mitochondrial aggregation ratio (n = 10 independent experiments). Scale bar, 20μm. (j-k) Representative images of MMP stained in CON, CON+Mel, SLE-M, and SLE-M+Mel group, and corresponding analysis (JC-1 red/green ratio; n = 10 oocytes per group). Scale bar, 20μm. (l-m) Representative images of ROS in MII oocytes after IVM from CON, CON+Mel, SLE-M, and SLE-M+Mel group, and quantitative fluorescence intensity (n = 10 oocytes per group). Scale bar, 50μm. (n-o) NADPH/NADP⁺, GSH/GSSG level in four groups (n = 3 independent samples). (p): Relative mRNA expression levels of ROS-related genes in MII oocytes after in vitro melatonin treatment (n = 3 independent samples). Data are presented as mean ± SD. Statistical analysis was performed using one-way ANOVA followed by Tukey’s multiple comparison test. *P < 0.05, **P < 0.01, ***P < 0.001.

**Supplementary Table1: RT–PCR Primers sequences**

| **Gene** | **Species** | **Forward primer (5′–3′)** | **Reverse primer (5′–3′)** |
| --- | --- | --- | --- |
| *Asz1* | mouse | CACGGCAGGGTCATAAAAATGT | CTCTTCTTTGGTTAGCTGCTGAA |
| *Casp1* | mouse | ACAAGGCACGGGACCTATG | TCCCAGTCAGTCCTGGAAATG |
| *Casp3* | mouse | ATGGAGAACAACAAAACCTCAGT | TTGCTCCCATGTATGGTCTTTAC |
| *Cat* | mouse | AGCGACCAGATGAAGCAGTG | TCCGCTCTCTGTCAAAGTGTG |
| *Dmc1* | mouse | ATGAAGGAGGATCAAGTTGTGC | CATGCTTCTGCAACAGGTCAA |
| *Gpx3* | mouse | CCTTTTAAGCAGTATGCAGGCA | CAAGCCAAATGGCCCAAGTT |
| *Gsr* | mouse | GACACCTCTTCCTTCGACTACC | CCCAGCTTGTGACTCTCCAC |
| *Meis2* | mouse | CTGGCGAGATCACGATGACG | AAGCTACGCTGTTGTCTAACC |
| *Mfn1* | mouse | CCTACTGCTCCTTCTAACCCA | AGGGACGCCAATCCTGTGA |
| *Ndc80* | mouse | GCCTCTCTATGCAGGAGTTAAGG | CGGTTTGTGTGTACTCAGCTT |
| *Nox4* | mouse | GAAGGGGTTAAACACCTCTGC | ATGCTCTGCTTAAACACAATCCT |
| *Nrf2* | mouse | GAGGAGAGAATTACTGCTTGGT | CCCTTTTTATTCCGGATTGCAA |
| *Prdx1* | mouse | AATGCAAAAATTGGGTATCCTGC | CGTGGGACACACAAAAGTAAAGT |
| *Shoc1* | mouse | CCCACAAGGGCAGAACTGAA | TGTGAACTCCTTCCGCAGTC |
| *Sod1* | mouse | AACCAGTTGTGTTGTCAGGAC | CCACCATGTTTCTTAGAGTGAGG |
| *Stat3* | mouse | CAATACCATTGACCTGCCGAT | GAGCGACTCAAACTGCCCT |
| *Tubb3* | mouse | TAGACCCCAGCGGCAACTAT | GTTCCAGGTTCCAAGTCCACC |
| *Tubg2* | mouse | ACAACTGGGGCAGAGGATTCT | GCAATGGAGTGACATAGCACA |
| *Gapdh* | mouse | GGTGAAGGTCGGTGTGACCG | CTCGCTCCTGGAAGATGGTG |

**Supplementary Table2**

| **Antibodies/** **Probe** | **Source** | **Identifier** |
| --- | --- | --- |
| α-Tubulin | Cell Signaling Technology | Cat # 3873T |
| OCT3/4 | Santa Cruz Biotechnology | Cat # sc-5279 |
| GPX4 | Proteintech | Cat #67763-1-Ig |
| Carboxy-H₂DCFDA | Beyotime | Cat # S0035M |
| MitoTracker Red | Beyotime | Cat # C1049B |
| JC-1 Assay Kit | Beyotime | Cat # C2006 |
| FerroOrange | Cell Signaling Technology | Cat # 36104S |
| BODIPY™ 581/591 C11 | Beyotime | Cat # S0043S |
| Monochlorobimane | MedChemExpress | Cat # HY-101899 |
| Hoechst 33342 | Beyotime | Cat # C1022 |
